# A human PBMC-based new approach method reveals PFAS-driven T-cell proliferation and immune dysregulation

**DOI:** 10.1007/s00204-026-04525-6

**Published:** 2026-08-25

**Authors:** Allison Loan, Lauren M. Bradford, Andrée Nunnikhoven, Gong Zhang, Emily Dupuis, Eunnara Cho, Matthew J. Meier, Rocio Aranda-Rodriguez, Azam Tayabali, Kristin M. Eccles, David Prescott

**Affiliations:** 1 Environmental Health Science and Research Bureau, Healthy Environments and Consumer Safety Branch (HECSB), Health Canada, Ottawa, ON K1N 6N5 Canada; 2 Bureau of Microbial and Toxicological Food Safety, Health Products and Food Branch, Health Canada, 251 Sir Fredrick Banting, Ottawa, ON K1A 0K9 Canada

**Keywords:** PFAS, Immunotoxicity, Cytokines, T-cell proliferation, NAMs

## Abstract

Immunotoxicity has emerged as a key health concern for per- and polyfluoroalkyl substances (PFAS), with animal studies showing reduced T-dependent antibody responses (TDAR) and epidemiological studies reporting decreased vaccine antibody titres. Notably, immunotoxicity is considered one of the most sensitive endpoints for PFAS exposure and has been used to inform regulatory guidance and health-based values. Given that more than 14,000 PFAS exist and are highly environmentally persistent, evaluating the immunotoxicity of individual compounds is critical but impractical, highlighting the need for efficient, human-relevant test systems that provide mechanistically informative, immune-relevant endpoints. Here, we assessed immunomodulatory effects of six PFAS analogues using an in vitro human peripheral blood mononuclear cell (PBMC) model. Two immune stimuli were applied, including lipopolysaccharide (LPS) to trigger innate responses and phytohemagglutinin (PHA) to simulate T-cell-mediated adaptive immune activation. Critically, several PFAS analogues enhanced T-cell proliferation following PHA activation, while a non-inclusive but overlapping subset of analogues suppressed cytokine secretion in response to PHA and LPS. Transcriptomic analyses indicate reduced B-cell identity and immunoglobulin gene expression alongside increased expression of genes associated with T-cell activation and proliferation. These findings implicate a dysregulated coordination between T- and B-cell responses as a potential mechanism underlying PFAS-associated immunotoxicity. Overall, the human PBMC model demonstrated that it is a cost-effective and ethical method for identifying and characterizing key events (KEs) in the adverse outcome pathway (AOP) for PFAS-induced immunotoxicity and has the potential to be refined and incorporated into a robust new approach method (NAM) to assess the next generation of commercial PFAS.

**Supplementary Information:**

The online version contains supplementary material available at https://doi.org/10.1007/s00204-026-04525-6.

## Introduction

Per and polyfluorinated alkyl substances (PFAS) are a class of human-made chemicals that have been widely used in the composition and manufacturing of several products across many industries. They possess extreme chemical and thermal stability, which has made them very stable in the environment (Glüge et al. 2020). Humans are exposed to PFAS through multiple pathways, including contaminated food and water, indoor dust, and everyday consumer products (Sunderland et al. 2019). Given the ubiquity and persistence of PFAS, it is critical to understand the health effects of PFAS exposure.

Some of the most sensitive indicators of PFAS-related health impacts pertain to alterations in immune system function, where a notable correlation between increased serum PFAS levels and decreased antibody titres to vaccines has been observed for a number of PFAS species (Grandjean et al. 2012; Granum et al. 2013; Crawford et al. 2023). Consistent with these observations, immune effects occur at lower exposure levels than many other adverse outcomes and are therefore considered among the most sensitive endpoints in PFAS risk assessment. Accordingly, the European Food Safety Authority (EFSA) set the tolerable weekly intake (TWI) levels for four of the most prevalent PFAS to 4.4 ng/kg BW/week for the sum of perfluorooctanoic acid (PFOA), perfluorononanoic acid (PFNA), perfluorohexane sulfonic acid (PFHxS), and perfluorooctanesulfonic acid (PFOS) (Bodin et al. 2020). This regulatory reliance on immunotoxicity underscores the need for mechanistically informed, human-relevant testing strategies.

In the early 2000s, a phase-out of PFOA and PFOS began among key manufacturers in the US. As a result of these activities, as well as the regulatory scrutiny on PFAS by countries worldwide, alternative shorter-chain PFAS began entering the market as replacements. For example, GenX, or hexafluoropropylene oxide dimer acid (HFPO-DA), has been introduced to the market as a replacement for PFOA. However, concerns have been raised about its safety and environmental persistence (Guo et al. 2024), and these safety concerns are not unique, as the US Environmental Protection Agency (US EPA) has identified over 14,000 potential PFAS compounds, which makes assessing PFAS-induced immunological impairments for each PFAS and PFAS-replacement compound time and resource-intensive (EPA 2022).

Traditional assessments of immunotoxicity have relied on i*n vivo* rodent studies. Specifically, the T-cell-dependent antibody response (TDAR) is a test that measures the rodent’s ability to generate antibodies following antigen exposure. Many rodent studies have consistently shown that exposure to PFAS suppresses the TDAR; however, the mechanisms underlying this suppression remain unclear (Keil et al. 2008; Peden-Adams et al. 2008; Loveless et al. 2008; DeWitt et al. 2009; Zheng et al. 2009). Antibody production requires coordinated interactions across a network of biological processes, including T-cell proliferation and activation, cytokine secretion, and B-cell maturation. Disruption at any node in this network could theoretically lead to reduced production of antibody titres. Current literature provides evidence that PFAS may alter cytokine secretion (Dong et al. 2011; Blais et al. 2026) and B-cell number and development (Taylor et al. 2023), though the extent to which each mechanism contributes varies across studies. Several underlying mechanisms have been proposed to explain these phenotypic changes, namely, modulation of NF-κB, peroxisome proliferator–activated receptors (PPAR), and calcium signaling (Ehrlich et al. 2023). However, the translation of this rodent data to inform on human endpoints is complicated due to species-specific differences in PPARα signaling and other nuclear receptor pathways, underscoring the need for human-relevant models (Nielsen et al. 2025). Assessing the immunotoxicity of numerous novel PFAS requires developing models that recapitulate in vivo endpoints and elucidate mechanisms of PFAS-induced immunotoxicity to support high-throughput assay development. The current body of literature examining the effects of PFAS exposure on in vitro assessments of adaptive immunity is relatively small, and while these studies collectively support the hypothesis that PFAS exposure is immunosuppressive by highlighting the ability of some members of this class to inhibit cytokine release, reduce the induction of cell surface markers indicative of immune activation, and alter key immune gene expression programs (Kasten-Jolly and Lawrence 2022; Janssen et al. 2023, 2024; Maddalon et al. 2023; Iulini et al. 2025a; b), gaps remain in our understanding of how PFAS affect the full scope of a complex adaptive immune response. These models and the information that they are designed to uncover can be directed by the application of an adverse outcome pathway (AOP) framework, in which exposure to a toxicant is linked to an ultimate biological outcome via a series of interrelated key events (KEs) (Ankley and Edwards 2018). Uncovering these KEs provides mechanistic insight into the biological processes underlying toxicity, which in turn allows us to develop refined tests that can inform hazard and risk assessment of individual chemicals.

To address these challenges and better align mechanistic assessment with human biology, we used an in vitro human peripheral blood mononuclear cell (PBMC) model to capture human-specific immune responses. We investigated the immunotoxicity of six PFAS analogues, including PFOS, PFOA, PFNA, perfluorodecanoic acid (PFDA), PFHxS, and PFAS replacement compound GenX. These compounds include both well-studied legacy PFAS with known immunological effects and less-characterized analogues. Additionally, the test compounds span a range of carbon chain lengths and include both carboxylate and sulfonate functional groups, which are commonly used to subgroup PFAS (ITRC 2023). PBMCs were exposed to PFAS, then stimulated to mimic environmentally relevant exposure scenarios. Two immune stimuli were applied, including lipopolysaccharide (LPS) to activate innate immune pathways, and phytohemagglutinin (PHA) to non-specifically activate the T-cell receptor (TCR) and simulate T-cell-mediated adaptive responses. Multiple endpoints were then measured, including cytokine secretion, T-cell proliferation, and transcriptomic profiling. By integrating cellular and molecular-level endpoints, our study aims to provide insight into immunological KEs that are impaired by diverse PFAS, and to begin to establish a mechanistically informed, human-relevant NAM to support immunotoxicity hazard identification and prioritization of PFAS.

## Methods

### PBMC and plasma acquisition

This research was approved by REB 2024-026 H. Purified PBMCs from 8 healthy adult donors (4 males and 4 females) were sourced from a commercial supplier (Table S1, Miltenyi Biotec, cat#150-000-572). Along with the PBMCs, corresponding plasma samples were also obtained for baseline PFAS analysis. Upon arrival, PBMCs were aliquoted in 10% dimethyl sulfoxide (DMSO, Millipore Sigma, cat#472301) and 90% heat-inactivated fetal bovine serum (FBS, Thermo Fisher, cat#A5256701) and stored in liquid nitrogen for future analysis. Corresponding plasma samples were also stored at − 80 °C for future analysis.

### Chemical preparation

All test chemicals were obtained in powder form, except for PFOS (Millipore Sigma, cat#77283, CAS#1763-23-1), and were prepared as concentrated stock solutions in their respective solvents. Stock solutions were prepared by dissolving each chemical in either methanol (Thermo Fisher, cat#A456-4) or DMSO, depending on the solubility of the compound. For GenX (Toronto Research Chemicals, cat#A634960, CAS#62037-80-3), methanol was used as the solvent due to its instability in DMSO (Liberatore et al. 2020), while PFOA (Millipore Sigma, cat#171468, CAS#335-67-1), PFOS (Millipore Sigma, cat#77283, CAS#1763-23-1), PFNA (Millipore Sigma, cat#394459, CAS#375-95-1), PFDA (Millipore Sigma, cat#177741, CAS#335-76-2), and PFHxS (Toronto Research Chemicals, cat#P999738, CAS#355-46-4) were dissolved in DMSO. To prepare stock solutions, the required amount of each chemical was weighed and dissolved in the appropriate solvent to achieve a stock concentration of 50 mM.

All chemicals were tested for endotoxin using the Pierce™ Chromogenic Endotoxin Quant Kit (Thermo Fisher, cat# A39553) according to the manufacturer’s instructions. Briefly, 50 mM stock solutions were diluted in the supplied endotoxin-free water to obtain 100 µM test samples. These samples were assayed alongside the low-range endotoxin standards (0.01–0.1 EU/mL) and read at 405 nm in duplicates. Since all samples fell below the standard curve, raw absorbance values are reported.

### Quantification of PFAS in the plasma

Plasma samples were thawed at room temperature. For all individuals, 100 µL of plasma was mixed with 10 µL of an internal standard (IS) mixture (25 ng/mL) and 890 µL of 60% methanol containing 2% formic acid, followed by 20 min sonication and micro solid phase (µSPE) extraction (Zhang et al. 2026; Blais et al. 2026).

The µSPE sample cleanup was conducted using 30 mg WAX µSPE cartridges (ITSP Solutions, cat#30P-WOWAX-T) with full automation via the PAL-RTC system (CTC Analytics). The µSPE cartridges were conditioned with 900 µL of 1% ammonia in methanol, followed by two washes with 600 µL of 60% methanol containing 2% formic acid. A total volume of 950 µL of acidified extract above was loaded onto the pre-conditioned cartridge at a rate of 5 µL/s, followed by two sequential washes dispensed at a rate of 20 µL/s: 900 µL of 60% methanol containing 2% formic acid, followed by 900 µL of 100% methanol. PFAS were eluted using 900 µL of 20 mM high-performance liquid chromatography (HPLC) grade ammonium acetate (Thermo Fisher, cat#A639-500) in methanol at a dispense rate of 10 µL/s. To prevent carryover and cross-contamination, the syringe was rinsed twice with methanol at full volume (1 mL) following sample loading and again rinsed once between each subsequent step. Eluates were then evaporated to dryness using a TurboVap system at 45 °C with a nitrogen flow rate of 0.7 mL/min. The dried extracts were reconstituted in 95 µL methanol and transferred to a 250 µL insert prior to LC-MS/MS analysis. Quantification of PFAS was performed using a TSQ Altis Plus triple quadrupole mass spectrometer (Thermo Fisher) coupled with a Vanquish ultra-performance liquid chromatography (UPLC) system (Thermo Fisher). A 5.0 µL aliquot of the sample extract was injected, and chromatographic separation was carried out at 30 °C on an ACQUITY UPLC BEH C18 column (1.7 μm, 2.1 × 50 mm; Waters, cat#186002350) equipped with a VanGuard BEH C18 pre-column (1.7 μm, 2.1 × 5 mm; Waters, cat#186003975). An isolator column (2.1 × 50 mm; Waters, cat#186004476) was installed between the mixer and the injection valve to act as a PFAS delay column. The mobile phases consisted of (A) 5 mM ammonium acetate in LC–MS water (Thermo Fisher) and (B) methanol. The UPLC gradient was performed at a flow rate of 0.2 mL/mins as follows: 5% B for 1.0 min, increased to 60% B over 1.0 min, ramped to 100% B over 6.0 min, held at 100% B for 2.0 min, returned to 5% B over 0.1 min, and held at 5% B for 4.8 min for column re-equilibration. The first 3 min of UPLC elutes were diverted to waste to remove potential coextracted polar components. Mass spectrometric detection was conducted using negative electrospray ionization (ESI) at a spray voltage of − 2.8 kV in selected reaction monitoring (SRM) mode. The ion transfer tube temperature, vaporizer temperature, sheath gas flow, and auxiliary gas flow were set to 325 °C, 350 °C, 50 AU, and 10 AU, respectively. All samples below the assay’s detection range were redefined as half of the detection limit. Detailed MS parameters and SRM transitions for individual target and their internal standard are listed in Table S2.

### PBMC thawing and seeding

For analysis, cryopreserved human PBMCs were rapidly thawed in a 37 °C water bath and resuspended in FBS with 100 U/mL micrococcal nuclease (S7 Nuclease) (Thermo Fisher, cat#EN0181) for 20s. This was followed by 5 mL of FBS.

### CFSE (carboxyfluorescein succinimidyl ester) staining

PBMCs were then immediately resuspended in 1 µM CFSE (Thermo Fisher, cat#C34554) solution and incubated for 20 min at 37 °C. Following staining, cells were resuspended in fresh phenol-red-free RPMI 1640 medium (Thermo Fisher, cat#11835030) supplemented with 10% heat-inactivated FBS and 1% penicillin-streptomycin (Thermo Fisher, cat#15140122) and seeded in a U-bottom 96-well plate at a density of 1 × 10^6^ cells/mL, in 200 µL, and left to rest overnight at 37 °C in 5% CO_2_. Cell number and viability were assessed using trypan blue.

### Chemical treatment

A 1:2.5 dilution of the 50 mM stock in culture medium was freshly prepared to yield a 20 mM working solution, which was further diluted 1:200 in the wells to obtain a final concentration of 100 µM. Subsequent PFAS concentrations were prepared by serial dilution of the 20 mM working solution using a diluent consisting of 60% culture medium and 40% DMSO to maintain a constant final DMSO concentration (0.2%) across all treatments.

Human PBMCs were exposed to 0, 0.5, 12.5, 25, 50, or 100 µM (Log_10_ + 1: 0, 0.18, 1.15, 1.41, 1.71, and 2.00) of PFOS, PFOA, PFNA, PFDA, PFHxS, and GenX 24 h prior to induction. The lower end of this concentration range was selected to approximate reported human plasma levels from the Canadian Health Measures Survey (CHMS, collection period: 2018–2019) (Health Canada 2023a). Appropriate vehicle controls (either DMSO or methanol) were included for each assay. For the 0 µM conditions, cells were exposed to an equal volume of the diluent (v/v). The DMSO or methanol concentration in culture was 0.2% of the media for all treatment groups.

After 24 h, at t = 0, human PBMCs were treated with 1 × (0.005 mg/mL) lipopolysaccharide (LPS, Thermo Fisher, cat#00-4976-93) or 1 × (0.0025 mg/mL) phytohemagglutinin-L (PHA, Thermo Fisher, cat#00-4977-03) by adding either LPS or PHA directly to the PFAS-containing well. Following induction at 24 h, 50 µL of the supernatant of each technical replicate was saved and pooled for enzyme-linked immunosorbent assays (ELISA) and frozen at − 80 °C. The same experimental cells were exposed to PFAS and immune stimulation for a total of 96 h to assess changes in proliferation using flow cytometry.

### Flow cytometry

Single cell suspensions were stained with LIVE/DEAD fixable aqua dead cell stain in PBS (1:1000, Thermo Fisher, cat# L34957) to exclude dead cells and debris, as per the manufacturer’s instructions. To block non-specific binding of antibodies by Fc receptors, prior to staining, cells were incubated with Mouse BD Fc Block (1:100, BD, cat#553141). Suspensions were then incubated with antibodies CD3 Monoclonal Antibody, APC (Thermo Fisher, cat#17-0037-42), and CD4 Monoclonal Antibody, eFluor™ 450 (Thermo Fisher, cat#48-0049-42) in flow cytometry Stain Buffer (BD Biosciences, cat#554715). Because CD3^+^/CD4^–^ T-cells in peripheral blood are predominantly CD8^+^ T-cells, with only a small population being CD3^+^/CD4^–^/CD8^−^, This population will be referred to as CD8^+^ going forward (Verschoor et al. 2015).

Data was acquired on Sony ID7000 Spectral Cell Analyzer, where a minimum of 5000 viable events were acquired per condition (based on fixable viability stain). Gates were set using fluorescence minus one (FMO) and single-color controls. The analysis was performed using FlowJo (v10.9.0). Since no sex-based differences were observed following flow cytometry and ELISA assay (assessed via two-way ANOVA), subsequent analyses were conducted on the combined dataset. Within each gated population, divided and undivided subsets were further gated with the LPS-only control used to define the undivided cell gate (within each donor), as LPS primarily stimulates non-T-cells and does not have a notable proliferative effect (Lawlor et al. 2021; Zhang et al. 2021). This gate was held constant across conditions, and cells outside of this gate (lower CFSE fluorescence) were considered divided (Fig. S3).

### Enzyme-linked immunosorbent assays (ELISAs)

Following induction at 24 h, 50 µL of the supernatant of each technical replicate was saved and pooled for ELISA and frozen at − 80 °C. Human IFN-gamma DuoSet ELISA (Bio-Techne, cat#DY285B), Human IL-2 Duoset ELISA (Bio-Techne, cat#DY202), and Human TNF-alpha DuoSet ELISA (Bio-Techne, cat#DY210) were performed according to the manufacturer’s instructions. For cytokine analysis, PBMC supernatant isolated 48 h post-PFAS exposure was diluted between 1:2 and 1:20 in reagent diluent, depending on the cytokine of interest. Absorbance was measured at 450 nm, with a reference wavelength of 540 nm used for wavelength correction. Raw absorbance values were converted to concentrations using a four-parameter logistic (4-PL) regression model generated from the standard curve. Samples were run in technical duplicate and averaged, then multiplied by their respective dilution factors to obtain concentrations (pg/mL). The assay detection range for IFN-γ was 9.38–600 pg/mL, while the detection ranges for IL-2 and TNF-α were 15.60–1000 pg/mL. If samples fell outside of the detection limit, they were re-run; any remaining samples below the assay detection limit were excluded from analysis.

To determine whether PFAS treatment impacted cell number, an XTT assay (Thermo Fisher, cat#X12223) was performed according to the manufacturer’s instructions. Briefly, cells were seeded in complete phenol-red-free RPMI 1640 medium in a U-bottom 96-well plate at a density of 1 × 10^6^ cells/mL in 100 µL per well. A seven-point standard curve of cell density was also prepared, ranging from 250,000 to 3906 cells/well, along with blank wells containing medium only. PFAS pre-treatments were performed as previously described, followed by stimulation with either LPS or PHA in technical duplicate. 48 h following chemical exposure (0.2% DMSO or 100 µM PFAS), XTT reagent was added, and the plates were incubated for 4 h at 37 °C. Absorbance was then measured at 450 and 660 nm. Cell density was determined by interpolation from the standard curve; samples that fell outside the curve range were retained in the analysis.

### Benchmark concentration and dose modeling

Cytokines detected in both the PBMC supernatant from this study and the mouse plasma from our previous in vivo study were selected for benchmark dose modeling (Blais et al. 2026). For in vivo data, PFAS dose values were assigned based on matched plasma concentrations (averaged within each condition) and converted from mg/L to µM using molecular weights for PFOS (538.12 g/mol) and PFOA (414.07 g/mol). Vehicle controls were assigned a nominal concentration of zero to anchor baseline responses. For the in vitro datasets, nominal PFAS concentrations were used.

Dose-response modeling was conducted using the *tcplfit2* package using the concRespCore function (Sheffield et al. 2022). Data was independently modeled for each cytokine, chemical, and dataset. Vehicle control values were summarized as median ± normalized median absolute deviation (nMAD), which were used to set benchmark response (BMR), background median (bmed) of the control, and cutoff thresholds (Nyffeler et al. 2020). Models were fit in a bidirectional and continuous hit-calling mode across multiple model types (e.g., cnst, hill, poly1/2, pow, exp2–5). The model with the lowest residual error among those that successfully converged was chosen as the winning model. Then, only winning models with hit probability ≥ 0.9, were able to fit lower and upper confidence limits of the points of departure (PODs) (PODL/PODU), and where the PODU/PODL ratio < 50 (Parham et al. 2025), were used for interpretation.

For visualization of raw and modelled dose–response curves, the *drc* package was used to fit four-parameter log-logistic models (LL.4) (Ritz et al. 2015). PODs derived from *tclpfit2* were overlaid to indicate modeled response thresholds.

### RNA sequencing

A separate experiment was performed for transcriptomic analysis. Here, human PBMCs were thawed and cultured in the presence of 100 µM (Log_10_ + 1: 2.00) PFOA, PFOS, or PFNA for 24 h, followed by treatment with 1X PHA for 72 h. The 72 h timepoint was selected to reflect transcriptional changes associated with T-cell proliferation, which typically precedes measurable phenotypic alterations. Total RNA was extracted from samples using the Qiagen RNeasy Mini Kit (Qiagen cat#74104) in combination with the QIAshredder spin columns (Qiagen, cat#79656) according to the manufacturer’s protocol. RNA concentration and purity were assessed using NanoDrop, and RNA integrity was evaluated by TapeStation. Only high-quality RNA (A260/280 ~ 2.0, RIN ≥ 9) was used for library preparation.

Libraries were prepared from high-quality total RNA in accordance with the Illumina Stranded mRNA Prep Ligation Kit (Document # 1000000124518 v04) (Illumina). The resulting libraries were quantified using the Qubit 3.0 Fluorometer – 1x dsDNA High Sensitivity Assay (Thermo Fisher) and validated using the Agilent 4200 TapeStation - D1000 Assay (Agilent). Libraries were then normalized to 2nM, pooled, and diluted to a final loading concentration of 750pM. Sequencing was performed using Illumina NextSeq™ 2000 P2 XLEAP-SBS™ Reagent Kit (100 Cycles). To generate sufficient read depth, two sequencing runs were completed.

Sequencing was carried out on an Illumina NextSeq™ 1000/2000 platform using two P2 flow cells with the XLEAP-SBS™ Reagent Kit (100 Cycles), which provides dual-indexing support and a maximum yield of ~ 400 million single reads per flow cell.

#### Sequencing data, quality control, and processing

Sequencing data were demultiplexed and converted into FASTQ format on the Illumina Basespace Sequence Hub using DRAGEN analysis v1.3.0 (Illumina). Processing and quality control of the FASTQ files, differential expression analysis, and exploratory statistical analyses were performed using the R-ODAF_Health_Canada pipeline (https://github.com/R-ODAF/R-ODAF_Health_Canada, downloaded August 25, 2025), which implements the Omics Data Analysis Framework for Regulatory application (Verheijen et al. 2022). The pipeline uses Snakemake (v8.0) (Mölder et al. 2021) and scripts (v.4.2.3) to manage workflows. Reads were trimmed using fastp (v0.23.2) (Chen 2025), then aligned against hg38 reference files (Dyer et al. 2025) using STAR (v2.7.10b) (Dobin et al. 2013). Expression levels were quantified with RSEM (v1.3.3) (Li and Dewey 2011), and a count matrix of genes per sample was produced. Across experimental samples, the median number of uniquely mapped reads was 28,264,905.

Quality control was performed to identify and remove outliers and low-quality samples, using Harrill et al. (2021) as a guideline. A Spearman’s correlation coefficient cutoff of 0.1 was applied across all experimental samples, and any samples clustering outside of this threshold were removed. The cut-off for uniquely mapped reads was set at 1 × 10^6^ per sample. Any samples outside of Tukey’s Outer Fence (3x interquartile range) for the following criteria were removed: the count of transcripts accounting for the top 80% of the signal, and the number of detected transcripts with at least five mapped reads. Samples with a Gini coefficient, an indicator of inequality in distributions, greater than 0.95 were excluded (Harrill et al. 2021).

The count matrix, including all experimental samples that passed quality control, were imported into R for statistical analysis. Following the R-ODAF guidelines (Verheijen et al. 2022), transcripts were filtered to retain only those for which at least 75% of the samples in an experimental group had counts above 0.5 counts per million (CPM). Additionally, spurious spikes were eliminated by excluding transcripts, where the difference between the maximum and median counts was less than the total counts divided by the number of replicates plus one.

Using DESeq2 version 1.38.0 (Love et al. 2014), differentially expressed genes (DEGs) were identified by comparing treatment groups to their respective vehicle controls within each sex, with donor ID included as a covariate in the DESeq2 formula. Shrinkage Log_2_ fold-change (Log_2_FC) was performed using the ashr method (Stephens, 2017). Results were extracted at an alpha threshold of 0.05 and reported as Wald test* p*-values, with false discovery rate (FDR) adjustment for multiple testing. DEGs were filtered using a Log_2_FC cutoff of 0.5 and an adjusted* p*-value (p_adj_) threshold of 0.05 for downstream analyses.

Data is available on NCBI’s Gene Expression Omnibus (GEO) under the accession number GSE317318.

#### Downstream analysis

DEGs were separated into upregulated (Log_2_FC > 0.5) and downregulated (Log_2_FC < − 0.5) subsets. Ensembl gene IDs were converted to Entrez IDs using *biomaRt*, and pathway enrichment analyses were conducted for Gene Ontology Biological Process (GO BP) and KEGG using the *clusterProfiler* packages. Enrichment significance was defined as *q-value* < 0.05. Pathways consistently enriched across all three PFAS contrasts, in at least one sex were identified and visualized.

### Statistical analysis

Figures and statistical analyses were performed in R (v4.3.2) (R Core Team 2023). Raw data were assessed for equal variance using Levene’s test and visually assessed for normality. Flow cytometry data were analyzed using a one-way repeated-measures ANOVA followed by Tukey’s multiple comparisons post hoc test. For statistical analysis, any replicate group for which one or more dose measurements were missing was excluded to maintain a balanced repeated-measures design. To confirm that any deviation from normality did not affect the outcome, results were additionally verified using a repeated-measures linear mixed model (LMM) with Tukey’s post hoc test, with both approaches yielding consistent conclusions. Following data filtering, the ELISA results contained several missing values across dose groups; therefore, analyses were conducted on raw data using a repeated-measures LMM, followed by Tukey’s post hoc test.

All flow cytometry and ELISA data, including points excluded from statistical analysis, are presented as normalized values relative to each donor’s corresponding vehicle control to facilitate comparison across groups. Chemical concentrations in figures are displayed on a Log_10_ + 1 scale to improve visualization across the tested concentration range while retaining the vehicle control group. Code is available at https://github.com/HC-EHSRB-CompTox/invitro-pfas-immunotox.

## Results

**Fig. 1:**
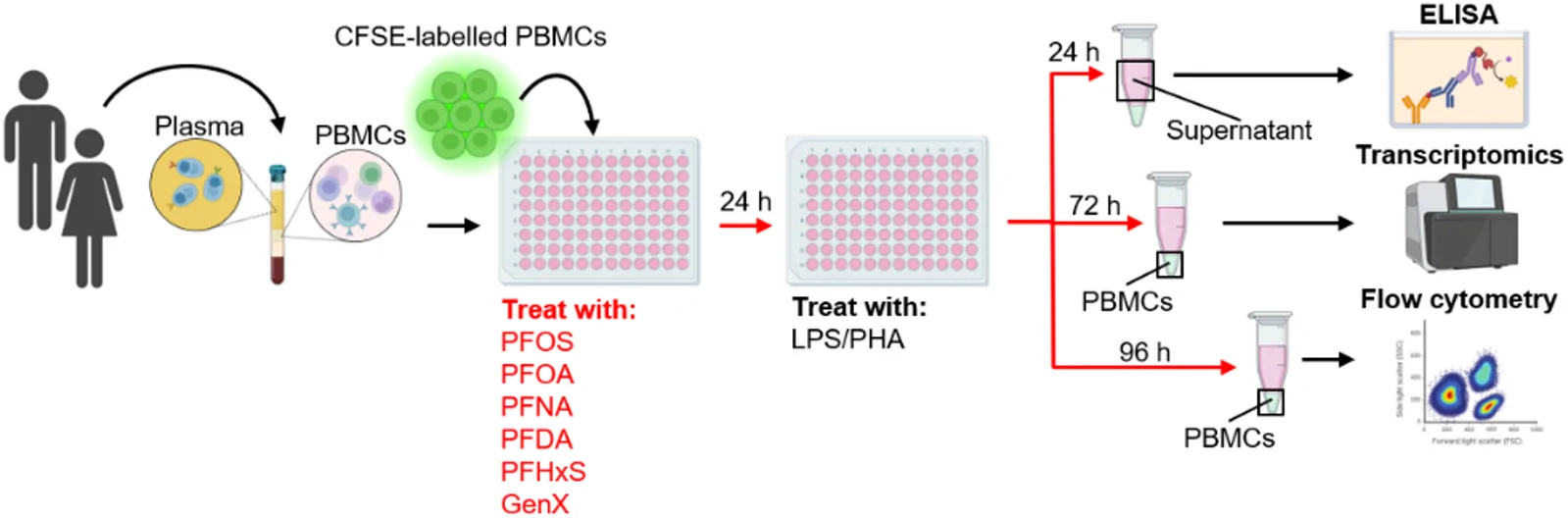
Experimental schematic, created in BioRender. Loan, (2026) https://BioRender.com/kmrwwon

To study the effect of PFAS on human PBMCs, we obtained PBMCs and corresponding plasma samples from 8 individuals, 4 males and 4 females, between the ages of 20 and 45 (Table S1). PBMCs were labeled with CFSE fluorescent dye to track cell proliferation and seeded in the presence or absence of PFAS. We examined 6 PFAS (PFOS, PFOA, PFNA, PFDA, PHFxS, and GenX) at 5 concentrations (0.5 µM, 12.5 µM, 25 µM, 50 µM, and 100 µM) in addition to a vehicle control (Fig. 1A). These PFAS were subsequently assessed for endotoxin contamination, which could artificially induce immune activation. All PFAS solutions tested were below the limit of detection of the standard (0.01 Eu/mL) and showed absorbance values comparable to a blank of endotoxin-free water (Fig. S1). Following PFAS pre-treatment for 24 h, cells were treated with either LPS or PHA. At 24 h post-LPS/PHA stimulation, the supernatant was isolated for downstream cytokine quantification. At 72 h, transcriptomic analysis was performed, and at 96 h, the PBMCs were processed for flow cytometry (Fig. 1).

**Fig. 2:**
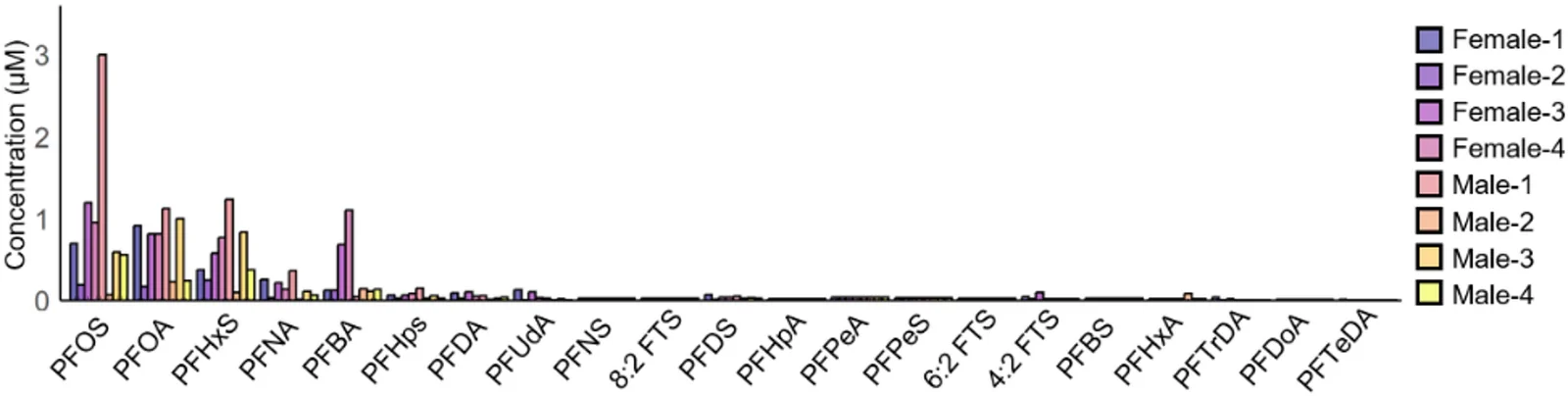
PFAS concentrations in the plasma of 8 donors. Coloured by individual. *n* = 8 humans

To ensure that none of the donors had been exposed to PFAS levels that could potentially skew the results, we measured 21 PFAS in the plasma via high-throughput LC-MS/MS, and detected 13 PFAS in at least 1 individual. The mean measured concentrations of the PFAS of interest were 0.9 µM, 0.66 µM, 0.15 µM, 0.05 µM, and 0.56 µM for PFOS, PFOA, PFNA, PFDA, and PFHxS, respectively. The measured background exposure of all donors was below the geometric mean of the Canadian Health Measures Survey (CHMS, collection period: 2018–2019) (Health Canada 2023a) and the geometric mean of the U.S. National Health and Nutrition Examination Survey (NHANES, collection period: 2017–2018) (Ale et al. 2024) (Figs. 2, S2). Consistent with this observation, previous studies have shown that frequent blood donation is associated with reduced PFAS levels (Honkanen et al. 2026). Therefore, exposure concentrations were not normalized to individual background levels.

### PFAS-induced T-cell proliferation

Flow cytometry was performed 96 h following stimulation to assess the proliferation of total T-cells (CD3^+^), CD4^+^ T-cells, CD3^+^/CD4^−^ (CD8^+^) T-cells, and non-T-cells (CD3^−^). Within each population, divided and undivided subsets were further gated. The LPS-only control was used to define the undivided cell gate, as LPS primarily stimulates non-T-cells, and does so without inducing proliferation (Lawlor et al. 2021; Zhang et al. 2021) (Fig. S3).

**Fig. 3:**
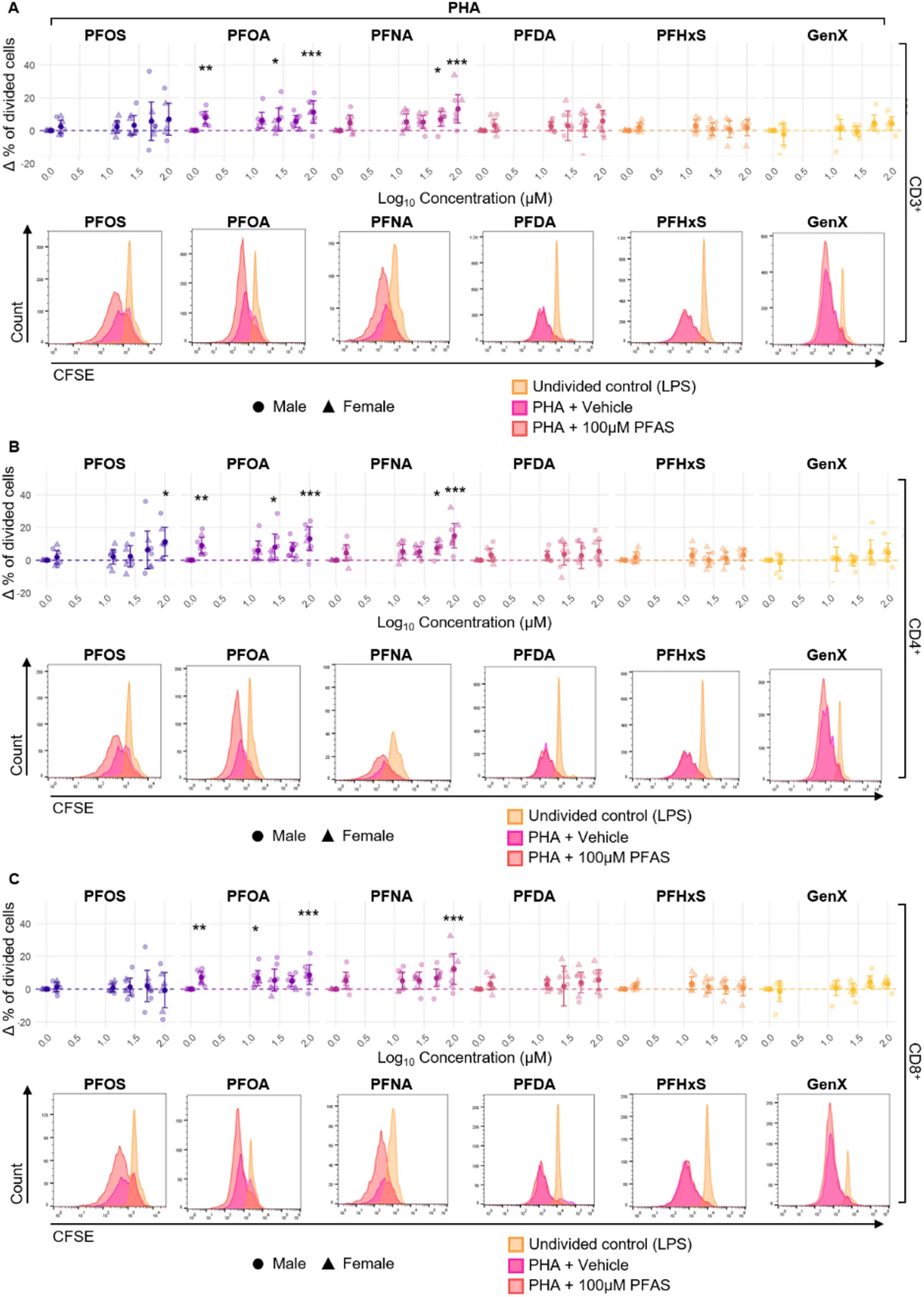
PFAS pre-treatment with PHA increases T-cell proliferation.** A**–**C** Proliferation of** A** CD3^+^,** B** CD4^+^, and** C** CD8^+^ T-cells following PHA stimulation was assessed by CFSE in the presence or absence of PFAS. Data are presented as Δ (treated − vehicle) ± 95% CI. Chemical concentrations are displayed on a Log_10_ + 1 scale. The dotted line represents the mean of the corresponding vehicle control. Representative density plots for PHA + Vehicle and PHA + 100µM (Log_10_ + 1: 2.00) PFAS compared to an undivided control. The shaded regions correspond to the distribution of CFSE intensity among cells for each PFAS tested. Statistical significance was assessed using the repeated measures one-way ANOVA with Tukey’s multiple comparisons (control: vehicle). *p ≤ 0.05; **p ≤ 0.01; ***p ≤ 0.001. *n* = 7–8 humans/group

To examine the role of PFAS treatment in combination with the T-cell activator PHA, we examined the percentage of divided CD3^+^, CD4^+^, and CD8^+^ T-cells and observed that treatment with PFOA + PHA and PFNA + PHA significantly increased the percentage of divided CD3^+^ T-cells, with effects observed at concentrations as low as 0.5 µM (Log_10_ + 1: 0.18) for PFOA and 50 µM (Log_10_ + 1:1.71) for PFNA (Fig. 3A). Within the CD4^+^ cell population, significant increases in cell division were observed following treatment with PFOS, PFOA, and PFNA, at concentrations as low as 100, 0.5, and 50 µM (Log_10_ + 1: 2.00, 0.18, 1.71), respectively (Fig. 3B). CD8^+^ T-cell division was significantly increased by PFOA and PFNA, with significant effects observed at 0.5 µM and 100 µM (Log_10_ + 1: 0.18 and 2.00), respectively (Fig. 3C). To further validate PFAS-induced proliferation, the reciprocal mean fluorescence intensity (MFI^−1^) of CFSE was used as an alternative measure. This method, which accounts for the number of cell division rounds a population undergoes, confirmed that all six PFAS induced some degree of proliferation (Pereira et al. 2020). PFOS, PFOA, and PFNA showed effects consistent with the results described above. Namely, PFOS increased proliferation only of CD4 + cells, whereas PFOA and PFNA increased proliferation of both CD4^+^ and CD8^+^ cells. Interestingly, MFI^−1^ analysis also revealed an increase in GenX-induced proliferation of CD8^+^ T-cells (Fig. S4). These findings suggest that MFI^−1^-based analysis may be more sensitive than binary division gating, as it captures the number of divisions rather than whether a single division occurred. These results suggest a potential difference between PFAS with sulfonate and carboxylate functional groups in their ability to promote T-cell proliferation, indicating structure-associated differences in immunomodulatory activity. Specifically, sulfonates like PFOS cause CD4^+^ T-cell proliferation as opposed to carboxylates like PFOA and PFNA, which cause broad CD3^+^ T-cell proliferation.

Since LPS primarily stimulates populations including monocytes, macrophages, dendritic cells, and B-cells, we next examined whether PFAS co-treatment altered CD3^−^, non-T-cell proliferation (Lawlor et al. 2021; Zhang et al. 2021). No changes in the percentage of divided CD3^−^ cells were observed following co-treatment with LPS in any PFAS tested (Fig. S5). No decrease in cell viability was observed following PFAS exposure at 96 h with either LPS or PHA stimulation (Fig. S6 A–B).

### PFAS-induced reduction of cytokine secretion

**Fig. 4:**
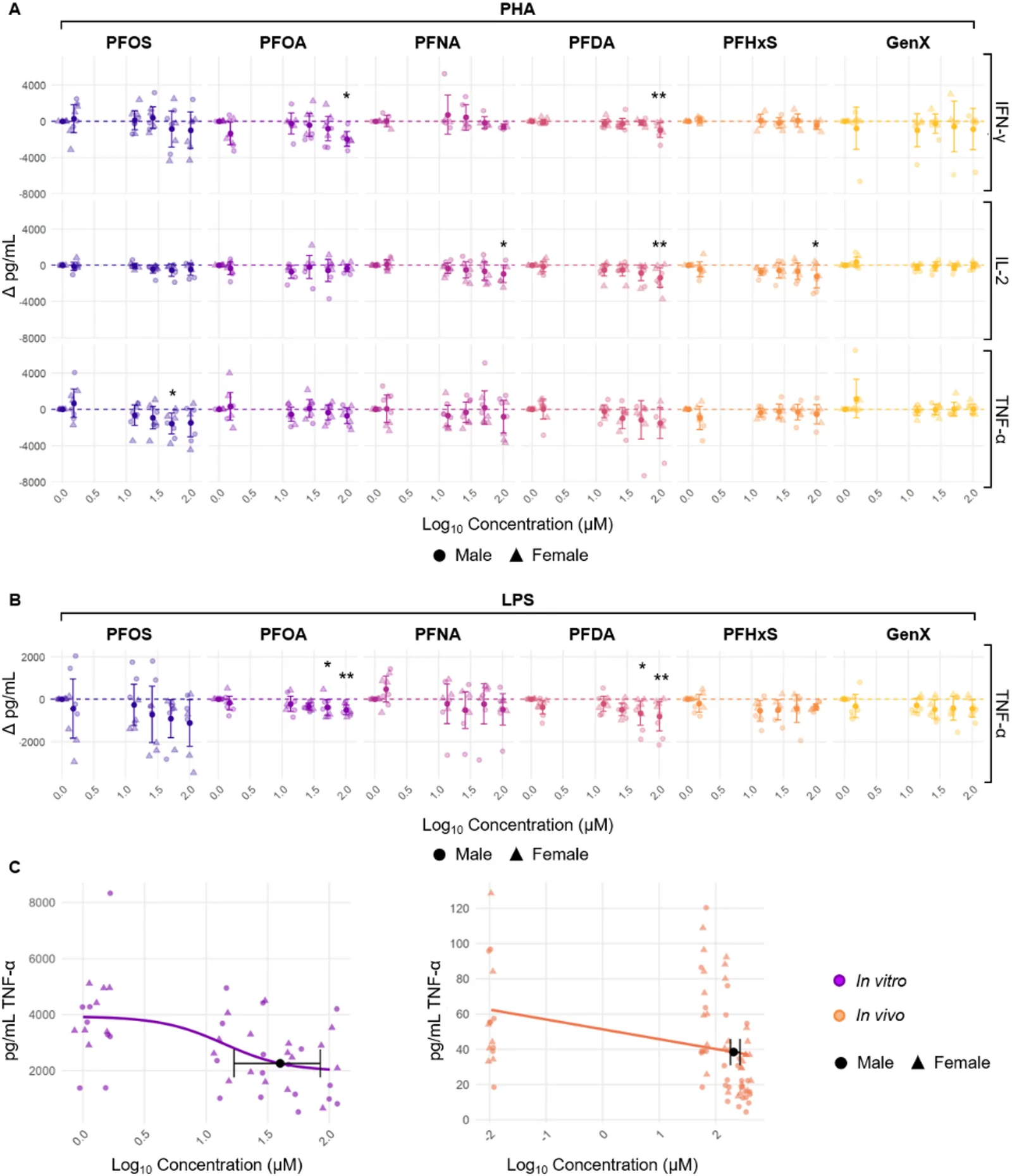
Pre-treatment with PFAS decreases cytokine secretion.** A** Concentration of IFN-γ, IL-2, and TNF-α in the supernatant 24 h after PHA treatment.** B** Concentration of TNF-α in the supernatant 24 h after LPS treatment. Data are presented as Δ (treated − vehicle) ± 95% CI. Chemical concentrations are displayed on a Log_10_ + 1 scale. The dotted line represents the mean of the corresponding vehicle control. Statistical significance was assessed using the repeated-measures LMM with Tukey’s multiple comparisons (control: vehicle). *p ≤ 0.05; **p ≤ 0.01. *n* = 7–8 humans/group.** C** Dose-response curve of TNF-α concentrations in the supernatant of PBMCs and plasma of experimental mice following PFOS exposure. Black dots and bars indicate PODs, PODL, and PODU. *n* = 7–8 humans/group

We analysed the effect of several PFAS on TNF-α, IFN-γ, and IL-2 secretion. These cytokines were chosen as they are produced by activated PBMCs and represent major components of T-cell-mediated immune function (Friberg et al. 1994). Co-treatment of PBMCs with PFAS and PHA revealed that cytokine secretion was reduced by several PFAS compounds. Specifically, PFOS significantly decreased TNF-α secretion at 50 µM (Log_10_ + 1: 1.71). PFOA significantly reduced IFN-γ levels at 100 µM. Both PFNA and PFHxS significantly reduced IL-2 at 100 µM, while PFDA decreased all three cytokines at 100 µM (Log_10_ + 1: 2.00) (Figs. 4A, S7A). To ensure that the observed changes in cytokine levels were not attributable to cytotoxicity, cell viability was assessed 24 h post-immune stimulation using an XTT assay at the highest PFAS concentration (100 µM). No difference in cell number was observed between 100 µM PFAS + PHA and DMSO vehicle + PHA, suggesting that overall cell number remained unchanged, while cytokine secretion was reduced (Fig. S7B).

In the LPS co-treatment groups, we found that only TNF-α release was detectable in the supernatant post-exposure. This was likely due to the low levels of IFN-γ and IL-2 known to be secreted from non T-cells, aside from those only found in very low proportions in the blood (De Groote et al. 1992; Yao et al. 1997). TNF-α secretion was significantly reduced at 50 µM (Log_10_ + 1: 1.71) following exposure to PFOA and PFDA (Figs. 4B, S7C). Similarly to the PHA-treated groups, no significant change in overall cell viability was observed across any LPS + PFAS treatment condition compared to 0.2% DMSO (Fig. S7D).

Previous work from our group treated C57BL/6 mice with 0.166, 0.5, 1, and 1.5 mg/kg BW/day of PFOS or PFOA for 56 days, followed by injection with sheep red blood cells (SRBCs) five days prior to euthanasia. We reported generally lower cytokine levels in the plasma of PFOS- and PFOA-exposed mice (Blais et al. 2026). To draw mechanistic parallels between our in vitro PBMC model and our established in vivo dataset, we derived comparative PODs. After filtering PODs as specified in the methods section, we identified a benchmark dose (BMD) of 206.67 µM for TNF-α following in vivo exposure to PFOS, and a lower benchmark concentration (BMC) of 39.87 µM from our in vitro PBMC model exposed to PFOS (Fig. 4C). However, we were unable to derive PODs for PFOA, PFNA, PFDA, PFHxS, and GenX exposures for IL-2 and IFN-γ, which failed to meet the hit call threshold, indicating insufficient evidence of a concentration-dependent response. This likely reflects that the highest concentration tested was not sufficient to produce a robust cytokine response.

### Transcriptomic analysis

**Fig. 5:**
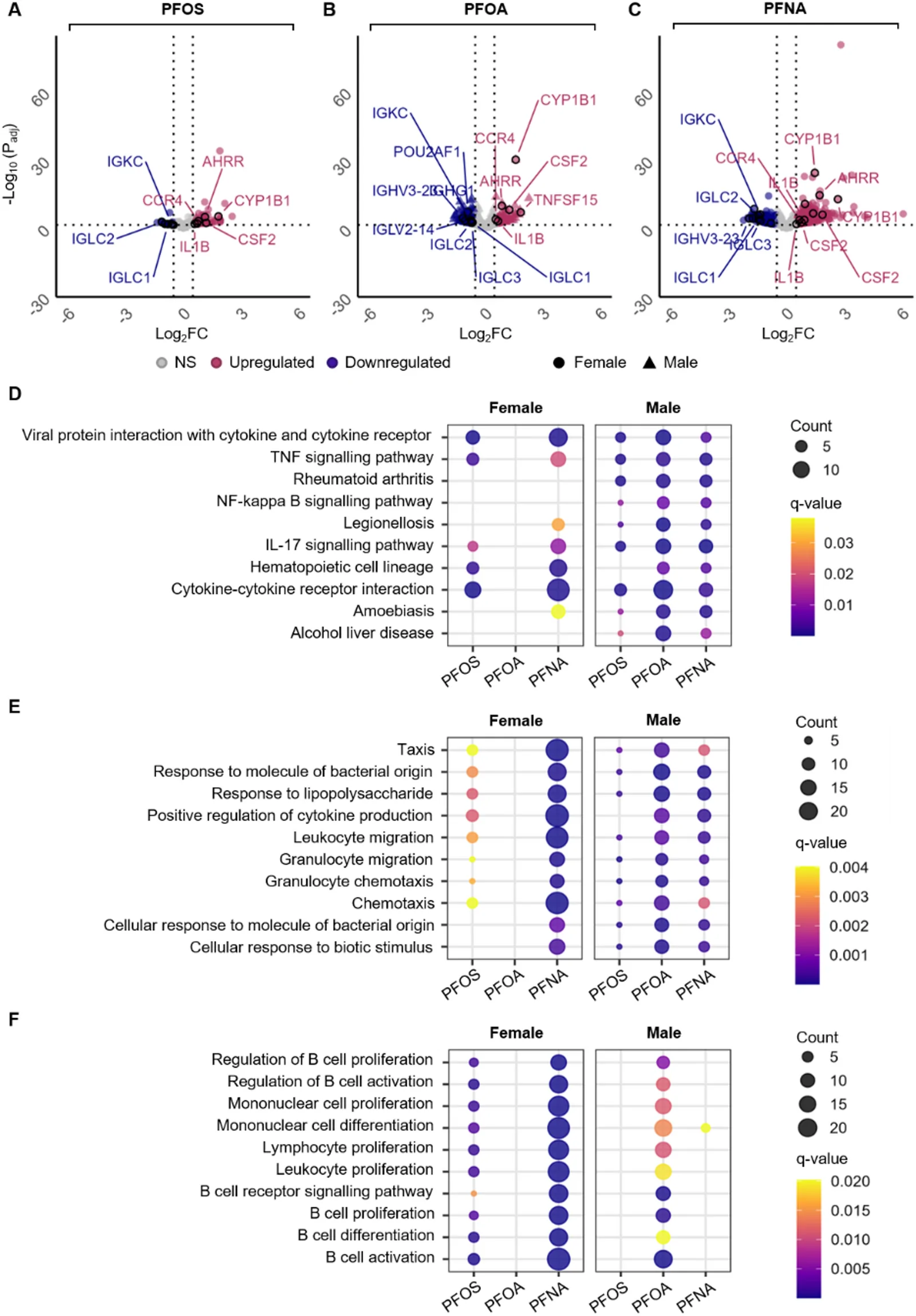
Transcriptional profiling of PFAS-exposed PBMCs reveals perturbations consistent with immune dysfunction.** A**–**C** Volcano plots showing differentially expressed genes (DEGs) between vehicle control (0 µM) and 100 µM (Log_10_ + 1: 0 and 2.00)** A** PFOS,** B** PFOA or** C** PFNA. DEGs were defined as those with Log_2_FC > 0.5 and p_adj_ < 0.05. Labelled genes represent select common DEGs across all three PFAS in at least one sex.** D**–**F** Dot plots showing the top 10 significantly enriched** D** KEGG pathways or** E**–**F** GO BP pathways (q-value < 0.05), sorted by average q-value across chemicals and sex. Dot plots are separated into (**D**–**E**) upregulated or** F** downregulated DEGs. Dot size reflects the number of DEGs in the pathway; colour indicates the q-value from enrichment analysis. *n* = 8 humans/group

To investigate potential mechanisms underlying these phenotypic and functional changes, transcriptomic analysis was performed on the PBMCs co-exposed with PHA and PFOS, PFOA, or PFNA. These PFAS were selected for more in-depth investigation due to their broad effects on T-cell proliferation and cytokine suppression. For the differential expression analysis, we treated the individual donor as a fixed variable, given that the PCA results showed greater similarity within individuals than within PFAS treatment groups (Fig. S8). Transcriptomics analysis revealed 53, 3, and 457 DEGs for females exposed to PFOS, PFOA, and PFNA, respectively. Exposure to the same chemicals in the males resulted in 9, 234, and 39 DEGs. Across all chemicals, several genes were shared in at least one sex, which are consistent with increased T-cell expansion. In particular, the elevated expression of *Ccr4* aligns with its known role in promoting Treg T-cell recruitment (Chiang et al. 2024), and increased expression of *Csf2*, *Mmp9*, and *Tnfsf15* is consistent with upregulation in activated T-cells (Migone et al. 2002; Fang et al. 2008; Benson et al. 2011; Li et al. 2013). Interestingly, many genes associated with B-cell identity and function are simultaneously downregulated. Namely, *Pou2af1*, a key gene regulating immunoglobulin secretion and B-cell maturation (Zhao et al. 2008), and several immunoglobulin genes, including *Igkc*, *Iglv2-14*, *Iglc1*, *Iglc2*, and *Iglc3* (Fig. 5A–C).

The KEGG and Gene Ontology Biological Process (GO BP) enrichment analyses identified 15 commonly upregulated KEGG pathways and 71 upregulated and 14 downregulated GO BP terms (Fig. 5D–F). Among the shared upregulated KEGG pathways, several were related to T-cell activation, including Viral protein interaction with cytokine and cytokine receptor (ID:04061), Cytokine-cytokine receptor interaction (ID:04060), IL-17 signaling (ID:04657), TNF signaling (ID:04668), NF-κB signaling (ID:04064), Hematopoietic cell lineage (ID:05410), and NOD-like receptor signaling (ID:04621) (Fig. 5D). Similarly, upregulated DEGs in GO BP were enriched for pathways associated with cellular movement, including taxis (GO:0042330), chemotaxis (GO:0006935, and cell chemotaxis (GO:0060326) among others (Fig. 5E). Interestingly, several GO BP terms related to bacterial responses were also enriched, including cellular response to biotic stimulus (GO:0071216), response to lipopolysaccharide (GO:0032496), cellular response to molecule of bacterial origin (GO:0071219), and response to molecule of bacterial origin (GO:0002237). Since all PFAS preparations were confirmed to be free of endotoxin (Fig. S1), it is unlikely that these pathways reflect true microbial stimulation. Instead, we hypothesize that these enrichments may result from overlap in gene functions between immune activation pathways.

Downregulated GO BP terms further support reduced B-cell identity and function, with significant enrichment of B-cell receptor signaling (GO:0050853), B-cell proliferation (GO:0042100), regulation of B-cell activation (GO:0050864), B-cell differentiation (GO:0030183), and regulation of B-cell proliferation (GO:0030888) (Fig. 5F). These findings provide transcriptomic evidence for the observed T-cell activation and concurrent suppression of B-cell function following exposure to PFOS, PFOA, and PFNA.

## Discussion

This study examined immunological alterations induced by PFAS analogues using an in vitro human PBMC model. By treating PBMCs with PFAS prior to stimulation, we aimed to mimic real-world human exposure scenarios. To probe immune function under diverse conditions, we applied two distinct immune stressors, LPS and PHA, representing innate and adaptive immune activation, respectively. This approach enabled us to assess how PFAS exposure modulates immune responsiveness. A range of immune endpoints, including cytokine secretion, T-cell proliferation, and transcriptomic changes, were used to comprehensively evaluate potential immune dysfunction.

Despite strong epidemiological and experimental evidence that PFAS exposure harms the humoral immune response, in vitro studies examining the mechanisms underlying this effect remain relatively understudied in comparison to rodent studies, which have consistently shown that exposure to PFAS suppresses the TDAR (Keil et al. 2008; Peden-Adams et al. 2008; Loveless et al. 2008; DeWitt et al. 2009; Zheng et al. 2009). This needs to be remedied, as a specific understanding of why this phenomenon occurs will allow us to design sensitive and high-throughput assays to determine whether replacements of legacy PFAS are similarly immunotoxic. Recent in vitro studies have demonstrated PFAS-mediated effects on B-cells, including decreases in the expression of antibody diversity-generating genes (Janssen et al. 2023), and decreases in total antibody production (Iulini et al. 2025a; b). However, the effects of PFAS on T-cell activation in vitro remain surprisingly understudied, given the central role of these cells in guiding the humoral response. The few studies that have been reported using primary human cells have established PFAS-induced alterations in cytokine release and surface marker activation phenotype (Corsini et al. 2011; Maddalon et al. 2023; Ayuk et al. 2025), indicative of immunosuppression. Kasten-Jolly and Lawrence reported a modest increase in proliferative response in both PHA- and influenza antigen-treated PBMCs exposed to lower concentrations of PFOA and PFOS (1–10 µM) in some, but not all, individuals assessed. This effect was reversed at the maximal dose 100 µM, where they observed a decrease in the proportion of proliferating T-cells from most donors, though this discrepancy can potentially be explained by their assessment of proliferation at the 7-day time point, at which sustained activation in the absence of IL-2 will have resulted in considerable activation-induced cell death (Kasten-Jolly and Lawrence 2022). Brieger et al. also observed a minor, non-significant decrease in proliferation in response to a different T-cell mitogen, concanavalin A, when PBMCs were exposed to PFOA and PFOS, as well as a slight increase in baseline proliferation when unstimulated PBMCs were similarly exposed (Brieger et al. 2011). Interestingly, Soloff et al. found a notable increase in the proliferative response of dolphin PBMCs exposed to PFAS in the absence of any mitogenic stimulation, suggesting that PFAS may be themselves mitogenic (Soloff et al. 2017). Taken together, it is clear that T-cell proliferative model design may affect the choice of stimulation, timing, and readouts, and this must be considered in future studies on PFAS.

While it may appear that increased T-cell proliferation is inconsistent with impaired vaccine responses, evidence suggests these phenomena can be mechanistically linked. T-cell proliferation kinetics are directly linked to TCR signal strength (Lewis et al. 2015), and it is well accepted that the strength and frequency of T-cell activation affects their fate, with strong, frequent signals leading to exhaustion or death (Qin and Xu 2025). This is particularly notable for developing T-cell precursors in the thymus, where strong TCR signals result in negative selection of thymocytes, as these developing T-cells are more likely to be self-reactive (Gascoigne et al. 2016). The strength of T-cell activation is also known to alter T helper differentiation into specific functional subsets, and thus, a change in TCR activity can ultimately lead to a skewing of the T helper repertoire away from the most efficient long-term antibody-producing program (Bhattacharyya and Feng 2020). For instance, high-dose antigen vaccines have been shown to produce less durable long-term antibody titres than lower, sustained antigen doses (Cirelli et al. 2019). It is therefore possible that higher circulating PFAS levels can paradoxically impair long-term immune response by promoting short-term T-cell proliferative activity. While this conflicts with the existing evidence that PFAS activate the PPAR family, which is generally thought to result in T-cell inhibition (Choi and Bothwell 2012), other known effects of PFAS, such as NF-kB activation (Wang et al. 2021) or the facilitation of glycolytic metabolism (Li et al. 2024) are associated with enhanced T-cell proliferative responses.

Our examination of proliferation revealed differences between PFAS species that may be explained by differences in chemical functional groups. While both PFOS and PFOA share an 8-carbon backbone, they differ in their functional group, as PFOS contains a sulfonate group, whereas PFOA features a carboxylate group. Other analogues can also be classified as such, with PFNA and PFDA also containing carboxylate groups, while PFHxS contains the sulfonate group (Health Canada 2023b). GenX, though structurally distinct, is a short-chain perfluorinated compound containing an ether bond and a carboxylate group (Guo et al. 2024). Proliferation results measured by MFI^−1^ suggest a potential difference between sulfonate and carboxylate-grouped PFAS in their ability to promote T-cell proliferation. Specifically, sulfonates such as PFOS promote CD4^+^ T-cell proliferation, whereas carboxylates such as PFOA and PFNA induce broad CD3^+^ T-cell proliferation. However, PFDA and PFHxS did not fit this grouping, suggesting that carbon chain length may also contribute to PFAS-induced proliferation. As reported by Iulini and colleagues, intracellular deposition of short-chain PFAS may be reduced, resulting in lower immunotoxic potential (Armitage et al. 2021; Iulini et al. 2025b). To this effect, GenX shows some similarities to legacy compounds but at a lower apparent potency, with only modest effects observed on cytotoxic CD8^+^ T-cell proliferation.

Despite the increase in T-cell proliferation at 96 h, PFAS treatment also suppressed several cytokines measured by ELISA at 24 h. While this may appear paradoxical, the timing of each measurement may largely influence the results. Cytokine production and its effects on the activation status of the immune cell milieu are highly dynamic and tightly regulated over time, and often controlled by active negative feedback loops (Carbone et al. 2024). Thus, if production of inflammatory cytokines is limited at an earlier time point, this may weaken the brake on the system that limits activation at later time points, resulting in a proliferative state that is sustained or augmented. For instance, while both TNF-α and IFN-γ initially facilitate T-cell proliferation in response to TCR ligation (Yokota et al. 1988; Siegel 1988) both cytokines are known to play a central role in activation-induced cell death (AICD), in which mature T-cells that bind MHC-presented antigen with excessive affinity are deleted in the periphery (Zheng et al. 1995; Refaeli et al. 2002). PHA stimulation is a well-established method of inducing AICD in vitro (Zhang et al. 2017), and it is possible that by limiting the initial burst of cytokine production, PFAS exposure is indirectly limiting the extent of AICD, which manifests as a perceived increase in proliferation. Similarly, IL-2 was originally designated as a T-cell growth factor due to its well-established promotion of T-cell differentiation and proliferation (Ross and Cantrell 2018), making it difficult to reconcile a decrease in IL-2 production with an increase in T-cell proliferation. However, IL-2 can also preferentially induce the expansion and activity of suppressive T regulatory cells (Li et al. 2018), which can produce factors that limit further proliferation.

Alternatively, other mechanisms of T-cell suppression could be altered by PFAS in a cytokine-independent manner that could result in increased T-cell proliferative capacity. For instance, hyperactivated T-cells can be induced into a state of exhaustion, partly mediated by metabolic reprogramming, where energy is diverted away from energetically costly functions such as rapid proliferation and cytokine production (Delgoffe and Powell 2015). Activated T-cells tend to favour energy production by aerobic glycolysis (Roos and Loos 1973), while exhausted T-cells limit it, partly via by the ligation of co-inhibitory receptors such as PD-1 on their surface (Patsoukis et al. 2015). Interestingly, PFAS have been shown to promote glycolysis in immune cells (Li et al. 2025), while the activation of PPARα has been shown to override T-cell exhaustion via the promotion of cellular energy metabolism (Chowdhury et al. 2018).

The immune alterations observed here mirror findings from our prior in vivo study (Blais et al. 2026), in which mice were exposed to PFOA or PFOS for 28 or 56 days, followed by sensitization with SRBCs five days before euthanasia. We observed PFAS-induced cytokine suppression alongside slightly elevated T-cell populations in the blood of some exposed groups, consistent with these in vitro findings. Comparison of the PODs derived in both studies revealed consistent trends in TNF-α suppression following PFOS exposure. Notably, PODs from the in vitro PBMC assays were generally lower than those from the in vivo study, suggesting greater sensitivity and highlighting the potential utility of this human-relevant in vitro model for early immune hazard identification. However, this association was observed only for PFOS at a single immunological endpoint, highlighting the need for further validation. Moreover, while both our in vitro and in vivo data show reduced cytokine secretion, other researchers have reported that PFAS-induced decreases in cytokine production are media-dependent. PBMCs exposed to PFOA or PFOS showed reduced IFN-γ secretion only in serum-containing media following stimulation (Kasten-Jolly and Lawrence 2022). Since our experimental conditions were conducted in serum-containing media, it remains unclear whether the observed cytokine suppression would be observed under serum-free conditions.

Transcriptomic analysis of PFOA-, PFOS-, and PFNA-treated PBMCs provided further evidence supporting the observation of increased T-cell proliferation. Several enriched KEGG pathways identified across PFAS compounds are associated with increased cytokine production. Moreover, increased expression of various genes across PFAS treatment conditions is associated with T-cell activation and recruitment, including *Ccl24*, *Ccr4*, *Csf2*, *Mmp9*, and *Tnfsf15*. Several of these genes are transcriptional targets of the NF-κB pathway, which was also identified as an enriched KEGG pathway across all three PFAS treatment groups. Our transcriptomics analysis also highlighted the potential role of B-cells following PFAS exposure. Immunoglobulins are a family of proteins produced by B-cells and plasma cells in response to antigen exposure. Critically, several immunoglobulin genes, including *Igkc*, *Iglv2-14*, *Iglc1*, *Iglc2*, and *Iglc3*, were downregulated across all PFAS compounds. Interestingly, each of these genes encodes for immunoglobulin light chains, whose transcription is regulated by B lymphocyte-specific transcription factors (Casellas et al. 2002). These changes in gene expression were further supported by enriched GO BP pathways, which also indicated decreased B-cell proliferation, differentiation, and activation. In vitro, decreases in B-cell populations have been observed in PBMCs exposed to PFAS mixtures following SARS-CoV-2 spike antigen stimulation (Ayuk et al. 2025). Downstream reductions in antibody production have also been observed in human PBMC models, which have shown that PFOA and PFNA significantly reduce anti-keyhole limpet hemocyanin (KLH) immunoglobulin M (IgM) production (Iulini et al. 2025a). However, other studies have found that PFOA and PFOS share limited similarity to known reference immunosuppressants based on bioactivity response profiles (Houck et al. 2023). In rodent models, a common endpoint observed using TDAR testing is decreased levels of IgM following PFOS and PFOA exposure (Keil et al. 2008; Peden-Adams et al. 2008; Loveless et al. 2008; DeWitt et al. 2009; Zheng et al. 2009; Dong et al. 2011). Similarly, in humans, select PFAS have been associated with decreased antibody titres in children (Grandjean et al. 2012; Granum et al. 2013; Crawford et al. 2023). Our findings contribute to the growing body of evidence that proposes that PFAS may influence B-cell activation or maturation (Taylor et al. 2023; Rudzanová et al. 2023). However, confirmatory analysis of decreased immunoglobulin levels should be performed in future studies for posterity. Although no sex differences were detected using ELISA or flow cytometry endpoints, transcriptomic analyses revealed sex-specific differences in the number of DEGs across several PFAS, suggesting increased sensitivity to sex-specific effects at the RNA level.

The interaction between PFAS and the NF-κB pathway has been well documented, with previous studies reporting both suppressed and heightened NF-κB activity following exposure (review, Ehrlich et al. 2023). In our study, we observed a similar response, where the enrichment of NF-κB signaling in KEGG pathway analysis using upregulated DEGs suggests PFAS-induced activation of this pathway. NF-κB is a central regulator of T-cell activation, integrating signals from cytokine receptors and costimulatory molecules to drive proliferation. Thus, the upregulation of NF-κB-related genes may explain the increased T-cell proliferation signatures observed across PFAS treatments. However, since we are examining a heterogeneous population, and NF-κB plays a role across multiple cell types, further investigation is required to confirm T-cell specificity. We also observed consistent upregulation of *Ahrr*, the repressor of the aryl hydrocarbon receptor (AhR), and *Cyp1b1*, both downstream transcription targets of AhR (Bock 2019). AhR is a xenobiotic-sensing transcription factor that engages in crosstalk with the NF-κB pathway and may amplify the transcription of inflammatory genes (Vogel et al. 2014). Critically, AhR activation regulates Th17 production; as a result, we hypothesize that dysregulated NF-κB and AhR activation may skew CD4^+^ T helper cell differentiation toward inflammatory Th1 or Th17 cells. Such a shift could reduce T follicular helper (Tfh) differentiation, thereby diminishing Tfh-dependent cues required for B-cell survival, class-switch recombination, and plasma-cell differentiation (Mohinta et al. 2015; Crotty 2019; Sheikh and Groom 2021). Depleted plasma-cell formation would ultimately reduce immunoglobulin secretion; however, targeted experiments directly assessing T-cell differentiation and B-cell maturation in the presence of PFAS will provide valuable opportunities to validate and further refine this proposed mechanism.

While this study represents an important advance toward developing a human-relevant, rapid test to assess immunotoxicity risk from chemicals such as PFAS, several limitations remain. First, B-cell identity was not examined beyond transcriptomic analysis. Direct phenotypic assessment is needed to determine whether PFAS exposure truly reduces B-cell numbers, or whether the apparent decrease is simply a relative effect driven by an increase in T-cell populations. Second, changes in immunoglobulin levels were not directly assessed, and future work should include confirmatory experiments to validate these findings. Finally, the absence of positive and negative immunomodulatory controls as well as a naïve treatment group limits the ability to fully contextualize the observed responses and constrains assay validation; this is a key limitation and should be prioritized in future work. Additionally, several next steps would strengthen these findings. First, T-cell differentiation should be evaluated to determine whether PFAS exposure shifts T-helper cell polarization toward a Th1 or Th17 phenotype and away from Tfh cell development. Second, qPCR validation of NF-κB and AhR signalling pathway involvement would add mechanistic support to the transcriptomic observations reported here. Finally, given that PFAS readily bind to lipids and serum proteins, reduced-serum or delipidated-FBS conditions should be explored to better characterize exposure dynamics in vitro.

Given that immunotoxicity represents a sensitive endpoint in PFAS risk assessment, scalable and human-relevant approaches such as the PBMC model described here may provide a useful proof-of-concept for hazard identification and for characterizing KEs within AOPs leading to TDAR suppression. Several AOPs associated with suppressed TDAR identify impaired cytokine signaling, including reduced IL-2 and IL-4 secretion, as a KE contributing to downstream immune suppression (Komatsu et al. 2023; Yasuhiro et al. 2025). Our findings suggest that altered T-cell proliferation may represent an additional cellular KE contributing to PFAS-mediated immune dysregulation. In support of this, the present study revealed four key findings. First, several PFAS analogues disrupted T-cell proliferation following PHA-induced immune activation to varying degrees of potency. Second, despite enhanced T-cell proliferation, PFAS exposure consistently suppressed cytokine secretion, suggesting complex, potentially dysregulated immune responses. Third, transcriptomics results indicate decreased immunoglobulin gene expression, consistent with rodent data, potentially indicating an immunosuppressive phenotype. Finally, this proof-of-concept study demonstrates that the human PBMC model may offer a cost-effective and ethical approach to evaluate the T-cell-specific immune effects of PFAS while providing an alternative to traditional animal testing, such as the in vivo TDAR assay. This platform additionally enables the incorporation of interindividual variability through the use of primary human donor cells.

## Supplementary Information

Below is the link to the electronic supplementary material.

Supplementary Material 1

Supplementary Material 2

## Data Availability

Transcriptomics data is available on NCBI’s Gene Expression Omnibus (GEO) under the accession number GSE317318. All other raw data were deposited in Mendeley at doi: 10.17632/v77vfbnz54.1.
