# Supplementary material for: A human PBMC-based new approach method reveals PFAS-driven T-cell proliferation and immune dysregulation
Source: Arch Toxicol. 2026 Aug 25;100(12):5451–68. doi: 10.1007/s00204-026-04525-6 (PMC13748203; doi:10.1007/s00204-026-04525-6)
Supplement: Supplementary file 1 [file 204_2026_4525_MOESM1_ESM.docx]

**Supplementary**

**Table S1. PBMC donor information**

| **Donor** | **Age** | **Smoker** | **Weight (kg)** | **Height (cm)** | **Race** |
| --- | --- | --- | --- | --- | --- |
| Female-1 | 43 | Yes | 53 | 167 | Caucasian |
| Female-2 | 29 | No | 91 | 173 | Caucasian |
| Female-3 | 41 | No | 61 | 168 | Caucasian |
| Female-4 | 22 | No | 195 | 174 | Caucasian |
| Male-1 | 42 | Yes | 95 | 183 | African American |
| Male-2 | 42 | No | 83 | 185 | Hispanic |
| Male-3 | 32 | Yes | 90 | 182 | Asian |
| Male-4 | 27 | Yes | 111 | 184 | Caucasian |

**Table S2. Compound name, acronym, CAS number, and key LC-MS/MS parameters for targeted PFAS and internal standard (IS), including IS choice, RF lens voltage, Selective Reaction Monitoring (SRM) transitions, and collision energy (CE).**

| **Compounds Name** | **CAS** | **Acronym** | **IS** | **RF (V)** | **Quantifier** | | **Qualifier** | |
| --- | --- | --- | --- | --- | --- | --- | --- | --- |
|  |  |  |  |  | **Transition** | **CE(V)** | **Transition** | **CE(V)** |
| Perfluorobutanoic acid | 375-22-4 | PFBA | MPFBA | 31 | 213.0>168.9 | 8.6 | NA | NA |
| Perfluoropentanoic acid | 2706-90-3 | PFPeA | M5PFPeA | 32 | 263.0>218.9 | 7.8 | NA | NA |
| Perfluorohexanoic acid | 307-24-4 | PFHxA | M5PFHxA | 37 | 313.9>268.9 | 8.3 | NA | NA |
| Perfluoroheptanoic acid | 375-85-9 | PFHpA | M4PFHpA | 43 | 363.0>318.9 | 9.1 | 363.0>168.9 | 17.2 |
| Perfluorooctanoic acid | 335-67-1 | PFOA | M8PFOA | 47 | 413.0>368.9 | 9.3 | 413.0>168.9 | 17.8 |
| Perfluorononanoic acid | 375-95-1 | PFNA | M9PFNA | 51 | 463.0>418.9 | 9.6 | 463.0>218.9 | 16.3 |
| Perfluorodecanoic acid | 335-76-2 | PFDA | M6PFDA | 56 | 513.0>468.9 | 10.4 | 513.0>218.9 | 17.9 |
| Perfluoroundecanoic acid | 2058-94-8 | PFUdA | M7PFUdA | 59 | 563.0>518.9 | 10.7 | 563.0>268.9 | 18.6 |
| Perfluorododecanoic acid | 307-55-1 | PFDoA | MPFDoA | 65 | 612.9>568.9 | 11.6 | 612.9>168.9 | 27.0 |
| Perfluorotridecanoic acid | 72629-94-8 | PFTrDA | MPFDoA | 73 | 662.9>618.9 | 12.1 | 662.9>168.9 | 28.2 |
| Perfluorotetradecanoic acid | 376-06-7 | PFTeDA | M2PFTeDA | 71 | 712.9>668.9 | 12.3 | NA | NA |
| Perfluorobutane sulfonic acid | 375-73-5 | PFBS | M3PFBS | 112 | 298.8>79.8 | 35.0 | 298.8>98.8 | 30.6 |
| Perfluoropentane sulfonic acid | 2706-91-4 | PFPeS | M3PFHxS | 126 | 348.8>79.8 | 40.3 | 348.8>98.8 | 34.7 |
| Perfluorohexane sulfonic acid | 355-46-4 | PFHxS* | M3PFHxS | 111 | 398.8>79.8 | 42.8 | 398.8>98.8 | 37.9 |
| Perfluoroheptane sulfonic acid | 375-92-8 | PFHpS | M3PFHxS | 161 | 448.8>79.8 | 46.1 | 448.8>98.8 | 40.8 |
| Perfluorooctane sulfonic acid | 1763-23-1 | PFOS* | M8PFOS | 120 | 498.8>98.8** | 44.6 | 498.8>79.8 | 51.6 |
| Perfluorononane sulfonic acid | 68259-12-1 | PFNS | M8PFOS | 250 | 548.8>79.8 | 57.1 | 548.8>98.8 | 48.4 |
| Perfluorodecane sulfonic acid | 335-77-3 | PFDS | M8PFOS | 119 | 598.8>79.8 | 57.7 | 598.8>98.8 | 50.8 |
| 4:2 Fluorotelomer sulfonate | 757124-72-4 | 4:2-FTS | M2-4:2 FTS | 101 | 327.0>306.9 | 19.7 | 327.0>79.8 | 35.7 |
| 6:2 Fluorotelomer sulfonate | 27619-97-2 | 6:2-FTS | M2-6:2 FTS | 124 | 427.0>406.8 | 23.1 | 427.0>80.8 | 34.9 |
| 8:2 Fluorotelomer sulfonate | 39108-34-4 | 8:2-FTS | M2-8:2 FTS | 120 | 527.0>506.8 | 26.8 | 527.0>486.8 | 33.0 |
| Perfluoro-n-(13C)butanoic acid | NA | MPFBA | NA | 31 | 217.0>172.0 | 8.6 | NA | NA |
| Perfluoro-n-(13C)pentanoic acid | NA | M5PFPeA | NA | 32 | 268.0>223.0 | 7.8 | NA | NA |
| Perfluoro-n-(1,2,3,4,6-13C)hexanoic acid | NA | M5PFHxA | NA | 37 | 318.0>273.0 | 8.3 | NA | NA |
| Perfluoro-n-(1,2,3,4-13C)heptanoic acid | NA | M4PFHpA | NA | 45 | 366.9>322.0 | 9.1 | NA | NA |
| Perfluoro-n-(13C)octanoic acid | NA | M8PFOA | NA | 47 | 421.0>376.0 | 9.3 | NA | NA |
| Perfluoro-n-(13C)nonanoic acid | NA | M9PFNA | NA | 51 | 472.0>427.0 | 9.6 | NA | NA |
| Perfluoro-n-(1,2,3,4,5,6-13C)decanoic acid | NA | M6PFDA | NA | 56 | 519.0>474.0 | 10.4 | NA | NA |
| Perfluoro-n-(1,2,3,4,5,6,7-13C)undecanoic acid | NA | M7PFUdA | NA | 59 | 570.0>525.0 | 10.7 | NA | NA |
| Perfluoro-n-(1,2-13C )dodecanoic acid | NA | MPFDoA | NA | 67 | 614.9>569.9 | 11.5 | NA | NA |
| Perfluoro-n-(1,2-13C )tetradecanoic acid | NA | M2PFTeDA | NA | 71 | 715.0>670.0 | 12.3 | NA | NA |
| Sodium perfluoro-1-(2,3,4-13C )butanesulfonate | NA | M3PFBS | NA | 113 | 302.0>80.0 | 31.6 | NA | NA |
| Sodium perfluoro-1-(1,2,3-13C)hexanesulfonate | NA | M3PFHxS | NA | 111 | 402.0>99.0 | 37.9 | NA | NA |
| Sodium perfluoro-1-(13C)octanesulfonate | NA | M8PFOS | NA | 121 | 507.0>99.0 | 45.6 | NA | NA |
| Sodium 1H,1H,2H,2H-perfluoro(1,2-13C) hexanesulfonate | NA | M2-4:2 FTS | NA | 101 | 329.0>309.0 | 19.7 | NA | NA |
| Sodium 1H,1H,2H,2H-perfluoro(1,2-13C) octanesulfonate | NA | M2-6:2 FTS | NA | 124 | 429.0>409.0 | 23.1 | NA | NA |
| Sodium 1H,1H,2H,2H-perfluoro(1,2-13C) decanesulfonate | NA | M2-8:2 FTS | NA | 120 | 529.0>509.0 | 26.8 | NA | NA |

* PFHxS and PFOS are mixtures that contain linear and a variety of branched isomers.

**Figure S1. Endotoxin in prepared chemical solutions.** Averaged raw absorbance values of technical duplicates measured for endotoxin in prepared PFOS, PFOA, PFNA, PFDA, PFHxS, and GenX solutions. Red dotted lines indicate the highest (0.1 EU/mL) and lowest (0.01 EU/mL) standard curve absorbance values.

**Figure S2. Baseline PFAS concentrations in human donor plasma compared to USA (NHANES) and Canada (CHMS) biomonitoring data.** Plasma concentrations of five PFAS of interest (PFOS, PFOA, PFHxS, PFNA, PFDA) in n=8 human donors. Data are coloured by individual. NHANES geometric mean (collection period: 2017-2018) and CHMS geometric mean (collection period: 2018-2019) reference values ± 95% CI.

**Figure S3. Flow cytometry gating strategy.**

**Figure S4. PFAS pre-treatment with PHA increases T-cell proliferation.** MFI^-1^ of CD3⁺, CD4⁺, and CD8⁺ T-cells following PHA or LPS stimulation was assessed by CFSE in the presence or absence of PFAS. Data are presented as mean MFI^-1^ fold-change, relative to vehicle. Chemical concentrations are displayed on a Log_10_ + 1 scale. Statistical significance was assessed using the repeated measures one-way ANOVA with Tukey’s multiple comparisons (control: vehicle). *p≤0.05; **p≤0.01; ***p≤0.001. n=7-8 humans/group.

**Figure S5. PFAS pre-treatment with LPS does not change non T-cell proliferation.** Proliferation of non-T-cells (CD3^-^) following LPS stimulation was assessed by CFSE in the presence or absence of PFAS. Data are presented as Δ (treated − vehicle) ± 95% CI. Chemical concentrations are displayed on a Log_10_ + 1 scale. The dotted line represents the mean of the corresponding vehicle control. Statistical significance was assessed using the repeated measures one-way ANOVA with Tukey’s multiple comparisons (control: vehicle). n=7-8 humans/group.

**Figure S6. PFAS pre-treatment followed by stimulation with PHA or LPS does not decrease cell viability.** (A-B) Percentage of live cells following (A) PHA or (B) LPS stimulation was assessed by fixable viability stain in the presence or absence of PFAS. Data are presented as Δ (treated − vehicle) ± 95% CI. Chemical concentrations are displayed on a Log_10_ + 1 scale. The dotted line represents the mean of the corresponding vehicle control. Representative histograms for the vehicle group show the distribution of fixable viability stain for each PFAS tested. Statistical significance was assessed using the repeated measures one-way ANOVA with Tukey’s multiple comparisons (control: vehicle). *p≤0.05; **p≤0.01. n=7-8 humans/group.

**Figure S7. Cytokine secretion shown in pg/mL and cell viability.** (A) Concentration of IFN-γ, IL-2, and TNF-α in the supernatant 24 h after PHA treatment. Data are presented as pg/mL ± 95% CI. Chemical concentrations are displayed on a Log_10_ + 1 scale. n=7-8 humans/group. (B) Number of cells/well following PHA stimulation for vehicle control (0.2% DMSO), 100 μM PFOS, 100 μM PFOA, 100 μM PFNA, 100 μM PFDA, 100 μM PFHxS, and 100 μM GenX-exposed cells. Data are presented as the number of cells/well ± 95% CI. The grey ribbon corresponds to the 95% CI of the DMSO vehicle control. n=8 humans/group. (C) Concentration of TNF-α in the supernatant 24 h after LPS treatment. Data are presented as pg/mL ± 95% CI. Chemical concentrations are displayed on a Log_10_ + 1 scale. n=7-8 humans/group. (D) Number of cells/well following LPS stimulation for vehicle control (0.2% DMSO), 100 μM PFOS, 100 μM PFOA, 100 μM PFNA, 100 μM PFDA, 100 μM PFHxS, and 100 μM GenX-exposed cells. Data are presented as the number of cells/well ± 95% CI. The grey ribbon corresponds to the 95% CI of the DMSO vehicle control. n=8 humans/group.

**Figure S8. CA shows donor-specific clustering of human PBMC responses.** PCA of vehicle control (0.2% DMSO), 100 μM PFOS, 100 μM PFOA, and 100 μM PFNA-exposed human PBMCs. Data points are coloured by individual donor.
