# Supplementary material for: A human PBMC-based new approach method reveals PFAS-driven T-cell proliferation and immune dysregulation
Source: Arch Toxicol. 2026 Aug 25;100(12):5451–68. doi: 10.1007/s00204-026-04525-6 (PMC13748203; doi:10.1007/s00204-026-04525-6)
Supplement: Supplementary file 2 [file 204_2026_4525_MOESM2_ESM.docx]

# Abbreviations

| **Abbreviation** | **Definition** |
| --- | --- |
| **4-PL** | four-parameter logistic |
| **4:2 FTS** | 4:2 fluorotelomer sulfonate |
| **6:2 FTS** | 6:2 fluorotelomer sulfonate |
| **8:2 FTS** | 8:2 fluorotelomer sulfonate |
| **AICD** | Activation-induced cell death |
| **AO** | adverse outcome |
| **AOP** | adverse outcome pathway |
| **AhR** | aryl hydrocarbon receptor |
| **BMC** | benchmark concentration |
| **BMD** | benchmark dose |
| **bmed** | background median |
| **BMR** | benchmark response |
| **CFSE** | carboxyfluorescein succinimidyl ester |
| **CHMS** | Canadian Health Measures Survey |
| **CPM** | counts per million |
| **DEG** | differentially expressed gene |
| **DMSO** | dimethyl sulfoxide |
| **EFSA** | European Food Safety Authority |
| **EPA** | Environmental Protection Agency |
| **ESI** | electrospray ionization |
| **FBS** | fetal bovine serum |
| **FDR** | false discovery rate |
| **FOSA** | perfluorooctanesulfonamide |
| **FSC-A** | forward scatter area |
| **FSC-H** | forward scatter height |
| **GEO** | Gene Expression Omnibus |
| **GO BP** | Gene Ontology Biological Process |
| **HPLC** | high-performance liquid chromatography |
| **IFN-γ** | interferon gamma |
| **IgM** | immunoglobulin M |
| **IL-2** | interleukin-2 |
| **IS** | internal standard |
| **KE** | key event |
| **KLH** | keyhole limpet hemocyanin |
| **LL.4** | four-parameter log-logistic models |
| **LMM** | linear mixed model |
| **Log_2_FC** | Log2 fold change |
| **LPS** | lipopolysaccharide |
| **MFI^-1^** | reciprocal mean fluorescence intensity |
| **N-EtFOSAA** | N-ethyl perfluorooctanesulfonamidoacetic acid |
| **N-MeFOSAA** | N-methyl perfluorooctanesulfonamidoacetic acid |
| **NAM** | new approach method |
| **NHANES** | National Health and Nutrition Examination Survey |
| **nMAD** | median absolute deviation |
| **p_adj_** | adjusted p-value |
| **PBMC** | peripheral blood mononuclear cell(s) |
| **PC** | principal component |
| **PFBA** | perfluorobutanoic acid |
| **PFDA** | perfluorodecanoic acid |
| **PFDoA** | perfluorododecanoic acid |
| **PFDS** | perfluorodecanesulfonic acid |
| **PFHpA** | perfluoroheptanoic acid |
| **PFHpS** | perfluoroheptanesulfonic acid |
| **PFHxA** | perfluorohexanoic acid |
| **PFHxS** | perfluorohexanesulfonic acid |
| **PFNA** | perfluorononanoic acid |
| **PFNS** | perfluorononanesulfonic acid |
| **PFOA** | perfluorooctanoic acid |
| **PFOS** | perfluorooctanesulfonic acid |
| **PFPeA** | perfluoropentanoic acid |
| **PFPeS** | perfluoropentanesulfonic acid |
| **PFTeDA** | perfluorotetradecanoic acid |
| **PFTrDA** | perfluorotridecanoic acid |
| **PFUdA** | perfluoroundecanoic acid |
| **PHA** | phytohemagglutinin |
| **PODL** | lower confidence bound |
| **PODs** | points of departure |
| **PODU** | upper confidence bound |
| **PPAR** | proliferator–activated receptors |
| **SRM** | selected reaction monitoring |
| **SSC-A** | side scatter area |
| **TCR** | T-cell receptor |
| **TDAR** | T-dependent antibody responses |
| **Tfh** | T follicular helper |
| **TNF-α** | tumor necrosis factor alpha |
| **TWI** | tolerable weekly intake |
| **UPLC** | ultra-performance liquid chromatography |
| **μSPE** | micro solid phase |
